# Topochemical Engineering of Cellulose—Carboxymethyl Cellulose Beads: A Low-Field NMR Relaxometry Study

**DOI:** 10.3390/molecules26010014

**Published:** 2020-12-22

**Authors:** Pieter De Wever, Rodrigo de Oliveira-Silva, João Marreiros, Rob Ameloot, Dimitrios Sakellariou, Pedro Fardim

**Affiliations:** 1 Bio- & Chemical Systems Technology, Reactor Engineering and Safety Section, Department of Chemical engineering, KU Leuven, Celestijnenlaan 200f, P.O. Box 2424, 3001 Leuven, Belgium; pieter.dewever@kuleuven.be; 2 Centre for Membrane Separations, Adsorption, Catalysis, and Spectroscopy for Sustainable Solutions, Department of Microbial and Molecular Systems, Celestijnenlaan 200f, P.O. Box 2454, 3001 Leuven, Belgium; rodrigo.deoliveirasilva@kuleuven.be (R.d.O.-S.); joao.marreiros@kuleuven.be (J.M.); rob.ameloot@kuleuven.be (R.A.); dimitrios.sakellariou@kuleuven.be (D.S.)

**Keywords:** cellulose, cellulose beads, hydrogels, NMR relaxometry, low-field NMR, surface energy, swelling, porous materials

## Abstract

The demand for more ecological, highly engineered hydrogel beads is driven by a multitude of applications such as enzyme immobilization, tissue engineering and superabsorbent materials. Despite great interest in hydrogel fabrication and utilization, the interaction of hydrogels with water is not fully understood. In this work, NMR relaxometry experiments were performed to study bead–water interactions, by probing the changes in bead morphology and surface energy resulting from the incorporation of carboxymethyl cellulose (CMC) into a cellulose matrix. The results show that CMC improves the swelling capacity of the beads, from 1.99 to 17.49, for pure cellulose beads and beads prepared with 30% CMC, respectively. Changes in water mobility and interaction energy were evaluated by NMR relaxometry. Our findings indicate a 2-fold effect arising from the CMC incorporation: bead/water interactions were enhanced by the addition of CMC, with minor additions having a greater effect on the surface energy parameter. At the same time, bead swelling was recorded, leading to a reduction in surface-bound water, enhancing water mobility inside the hydrogels. These findings suggest that topochemical engineering by adjusting the carboxymethyl cellulose content allows the tuning of water mobility and porosity in hybrid beads and potentially opens up new areas of application for this biomaterial.

## 1. Introduction

Emerging applications such as (bio-)catalyst design [1], cell harvesting [2,3], tissue engineering [4] and (bio-)sensor development [5,6] demand the more advanced engineering of current hydrogel and aerogel structures, such as cellulose-based gels. Cellulose derivatives represent an abundant, biodegradable supply of renewable biopolymers suitable for both multi-functionalization and shaping into optimized functional materials, for instance, microspheres and beads. Cellulose hydrogels, in particular, can be fabricated through environmentally friendly processes, sustaining an increasing interest in cellulose for numerous applications, for example, adsorbent preparation, enzyme immobilization supports, and drug loading and delivery matrices [7].

A multitude of shaping techniques have since arisen to shape polysaccharides into highly engineered spherical structures. Such processes commonly rely on one of two methods: (1) the mixing of a polymer solution with an immiscible solvent to generate micrometer-sized droplets that are subsequently precipitated, or (2) dropping a solution of dissolved polysaccharides into a non-solvent to initiate coagulation [8]. In the latter approach, rapid initial skin formation at the cellulose/non-solvent interface is common [7]. This boundary skin or “shell” acts as a membrane with an inherently different structure from the bead’s core [9]. Despite this fact, the structure of these cellulose beads remains sensitive to drying conditions [10]. Considerable shrinkage and a loss of porosity are common upon water removal, highlighting strong interactions between bead-bound water and the cellulose matrix [11]. Notwithstanding numerous studies, a considerable knowledge gap remains. As a result, a growing interest in a better understanding of water–cellulose interactions in these materials has been observed in recent years [12,13,14].

The water–cellulose interaction has been outlined as instrumental in the definition of the structural and morphological properties of shaped cellulose. According to Caulfield [15], this interaction corresponds to a surface phenomenon where both material properties and geometry play a crucial role. Concerning the hydrogel’s geometry, two main factors have been identified: the cellulose fibril interaction with water (microstructure) and the influence of water on the larger lamellar structure (meso-/macrostructure). Current models suggest both amorphous and Iα cellulose swell in water since the solvent can wet and even chemically interact with the cellulose fiber bundles. However, water is unable to disrupt the strong attraction between individual cellulose chains [12,13,14]. Nonetheless, it has been demonstrated that the use of additives, such as NaOH and urea, can promote cellulose dissolution through the disruption of the hydrogen bonds, electrostatic interactions, and van der Waals dispersion forces present in water–cellulose systems. Trygg and coworkers used NaOH–urea–water to dissolve cellulose, followed by precipitation in an antisolvent. Pristine cellulose beads were then fabricated with surface areas over 300 m^2^/g [16]. The mercerization changes the cellulosic structure, leading to the formation of cellulose II [17], a cellulose polymorph with different water interactions [18].

Despite the interest in these highly engineered hydrogels, not much is known in regard to the water interaction with blended polysaccharide matrices with a complex internal hydrogel geometry. The topic is particularly vital for protein and cell studies, as these are greatly affected by their immediate microenvironment, in which water interactions play a vital role [19]. In (immobilized) proteins, the presence of water can change the hydration and the protein dynamics [20,21]. Bound water, in particular, has been shown to have a stabilizing effect on proteins, reducing their denaturation [20].

In this work, we applied a topochemical engineering approach, shaping blends of cellulose and carboxymethyl cellulose into a designed core–shell structured macrosphere. The effect of integrating carboxymethyl cellulose into cellulosic bead structures was investigated as a means of tuning the pore architecture and water–bead interactions. Low-field nuclear magnetic resonance (NMR) relaxometry was used to examine surface–water interactions, as well as to evaluate the water mobility through hydrogen relaxation times (T_1_ and T_2_). This technique has already shown success in monitoring changes in the water accessibility of lignocellulose subjected to different alkali and acid pretreatments [22,23], in the determination of pore sizes in pure cellulose beads [24], and in expressing tortuosity in polyacrylamide hydrogels [25].

## 2. Results and Discussion

A series of cellulose–carboxymethyl cellulose (CMC) composite beads containing 0–30% (*w*/*w*) CMC was prepared via drop formation and coagulation in an antisolvent (Table 1). CMC, a polyelectrolyte known to promote the formation of supramolecular assemblies with cellulose by means of hydrogen bonds between both species [26], was selected as a secondary polymer for the fabrication of anionic hybrid beads. The process consisted of the dissolution of cellulose and CMC in solutions of water–NaOH–urea between −9 and −12 °C. These mixtures were kept under stirring, leading to the formation of murky solutions due to the entrapment of air bubbles. The bubbles were, however, removed through a supplementary step of centrifugation, yielding transparent solutions. A correlation between the amount of CMC added and the size of the particles formed was observed. At low CMC concentrations, distinct droplets were formed that resulted in well-defined spherical particles. Beads with a higher polyelectrolyte content saw a rise in droplet diameter. At high CMC concentrations, the droplets exhibited significant tail formation (e.g., Cel30) upon detaching from the nozzle due to the increased viscosity of the solution. Particle asymmetry was mitigated through the adjustment of the nozzle height and use of low flow profiles to facilitate droplet detachment from the nozzle.

During coagulation in acid, both cellulose and carboxymethyl cellulose precipitate. Low-pH conditions, below the isoelectric point of CMC, cause an enhancement of the CMC’s affinity towards cellulose. The precipitated particles were collected and rinsed to neutrality, a process reported to induce the partial leaching of surface-bound CMC [27]. The conjoint dissolution of the anionic polyelectrolyte and cellulose results in the formation of a “composite” structure in which CMC is well-integrated in the generated cellulose network. Still, the CMC on the beads’ outer surface may be easily detached. Our study made use of CMC with an intermediate molecular weight, as the molecular weight does not affect the surface charge, and this choice minimizes the downsides of both high- and low-molecular-weight CMC [28]. CMC with a high molecular weight forms highly viscous solutions that impair droplet formation but has been shown to promote the greater swelling of cellulose fiber surfaces, due to the formation of elongated brush-like structures. However, these structures are absent for low-molecular-weight CMC systems [29].

The specific surface area of the pristine cellulose beads is comparable to the areas obtained by Trygg and coworkers, in which the bead architecture was designed through the experimental optimization of parameters such as the pulp quantity added, coagulation bath temperature, and acid concentration [16]. The work presented here introduces a fourth parameter by mixing two polysaccharides to form a hybrid bead, while keeping the total polysaccharide mass constant. Table 1 depicts the effect of CMC incorporation in the beads’ architecture (i.e., chemical and structural identity). Increasing the CMC content lowers the available surface area from 419 m^2^/g down to 311 m^2^/g, while increasing the total pore volume, expressed as the porosity, and particle swelling. The wetting of beads prepared with 30% (*w*/*w*) CMC saw an enhancement factor of 4.65 for swelling, compared to pristine cellulose beads, demonstrating highly efficient water sorption for the hybrid material. Typically, carboxymethyl cellulose-based hydrogels display a high sorption capacity, desirable for ecological superabsorbent applications [30,31,32]. A comparison of the hybrid hydrogel beads prepared in this paper revealed lower swelling compared to the cross-linked CMC–cellulose sheets prepared by Salleh et al. [33] and Chang et al. [34], an effect ascribed to the higher cellulose content in the herein-reported beads, as well as the different gel fabrication process employed.

**Figure 1:**
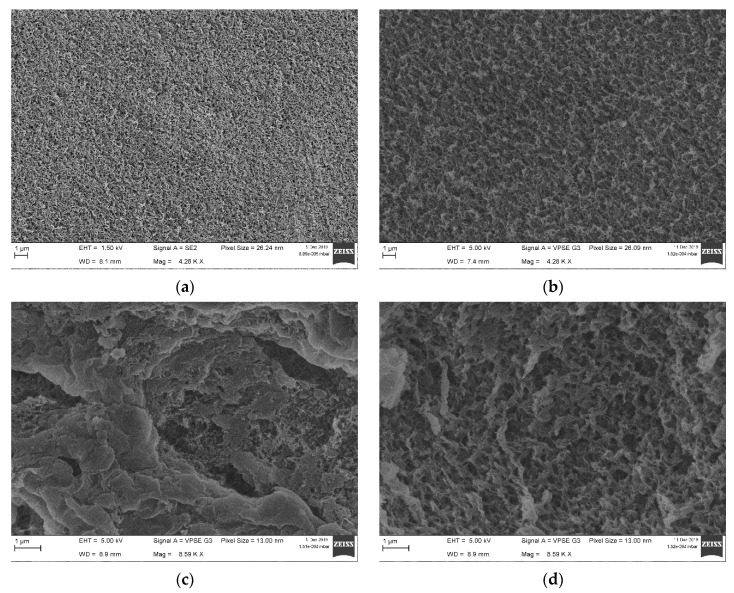
FE-SEM images of surfaces (**a**,**b**) and cross-sections (**c**,**d**) of Cel0 (left) and Cel30 (right) beads. CMC addition increases pore sizes on the surface and inner regions of the beads.

Field emission scanning electron microscopy (FE-SEM) images of the critical point dried (CPD) samples (Figure 1) illustrate the surface morphology (Figure 1a,b) and the cross-section (Figure 1c,d) of the pristine and hybrid cellulose–CMC beads, respectively. The addition of CMC favors an increase in pore size, at the particle surface, while the cross-sectional images reveal the formation of pores larger than on the surfaces. The differences in porosity between the surface and core sections are likely the result of CMC–cellulose coagulation under the acid bath conditions, which promotes the fast formation of a boundary shell/skin with a thickness in the range 1–10 µm. This process takes place as soon as the droplet comes into contact with the antisolvent. Once solidified, the formed shell likely acts as a barrier, hindering mass transport between the external solution and the inner medium (liquid core). As a consequence, a secondary structure is formed in the inner section of the beads, as demonstrated in the work of Fan et al. [9], who studied the formation of cellulose fibers over time. The aforementioned study also demonstrates the formation of a distinct core–shell structure based on diverse diffusion mechanics.

Increased porosity, on both the surface (Figure 1b) and core (Figure 1d) domains, likely occurs due to the presence of carboxyl groups, as well as ion exchange between Na and H during the coagulation step. As expected, the resulting increase in average pore size, from mesopores to macropores, yields a considerable reduction in the BET surface area (Table 1). The pore architecture of cellulose–CMC beads reported here displays a regular morphology, contrasting with results from Chang et al. [34] and Salleh et al. [33], who documented large microcavities in the respective cellulose–CMC gels.

**Figure 2:**
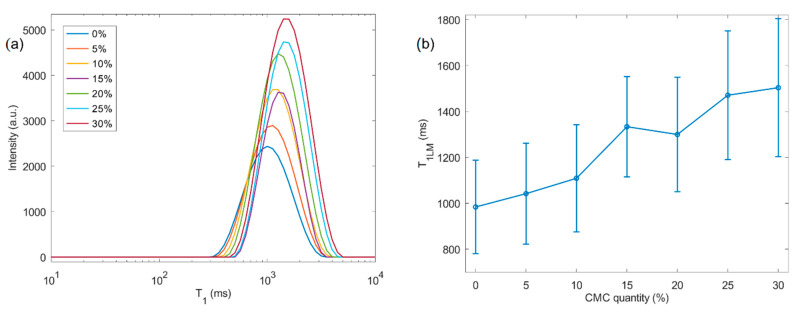
^1^H NMR longitudinal relaxation measurements. (**a**) T_1_ time distributions from the cellulose-based samples for various % of CMC (in legend), showing an increase in both intensity and T_1_ relaxation time. This is also shown in (**b**) by plotting the log-mean T_1_ values, calculated from the T_1_ distributions, as a function of CMC percentage. Error bars indicate the width of the distribution.

The complementary characterization of the produced beads was carried out with recourse to low-field NMR relaxometry. In Figure 2a, the ^1^H NMR longitudinal relaxation time distributions (T_1_) are presented for different CMC contents, ranging from 0 to 30% (*w*/*w*). A clear increase in both the average T_1_ value (Figure 2b) and distribution width were recorded in direct proportion to the CMC doping amount. A clear trend is observed in which the determined distribution fits for T_1_ consistently shift to higher values with the amount of CMC present in the hybrid beads.

We observe that the average T_1_ increases with swelling for beads that are composed of two blended polysaccharides. The longitudinal nuclear relaxation is related to the energy exchange between the adsorbed water and the surrounding material. As such, two distinct hypotheses may account for the observed changes in the T_1_ results: (1) an increase in water–pore wall interaction strength, and (2) a reduction in the effective “binding” of water molecules in the pore walls due to swelling. In the former, water molecules would interact strongly with the material, exhibiting solid-like behavior, as demonstrated by the Bloembergen–Purcell–Pound (BPP) model [35], while in the latter, bead swelling would lead to a reduction in the effective confinement of water molecules, which would converge towards the corresponding bulk state (capillary condensation). Pure gels from carboxymethyl cellulose [36] and cellulose ethers [37], e.g., hydroxyethyl, hydroxypropyl and hydroxypropyl methylcellulose, demonstrate an increase in T_1_ with water content.

Courtenay et al. [38] changed the degree of substitution (DS) in cationic cellulose gels. They reported an inverse relationship between the DS and T_1_ relaxation times. Our study, however, revealed an increase in T_1_ relaxation time for beads richer in CMC (Figure 2b). In light of this observation, we expect that bead swelling primarily impacts the energy exchange of the protons.

**Figure 3:**
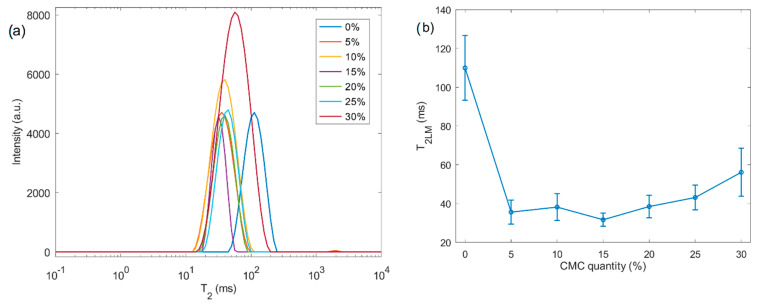
^1^H NMR transverse relaxation results. (**a**) T_2_ distributions of cellulose-based samples. values for the pristine cellulose (sample Cel0); the T_2_ was shortened with the addition of various percentages of CMC (shown in the legend). (**b**) Log-mean of T_2_ distributions showing a sudden shortening in value (5% *w*/*w* CMC), followed by an incremental transition towards higher values with increasing the amount of CMC. Error bars indicate the width of the distribution.

Transverse relaxation time (T_2_) NMR measurements were also performed. The results are displayed in Figure 3. The fitted T_2_ distributions (Figure 3a) revealed an abrupt shift towards lower T_2_ values upon the introduction of a minimal amount of CMC (5% *w*/*w*), followed by a progressive transition to higher times, as further presented in Figure 3b. An initial sharp drop in the log-mean transverse relaxation time (T_2LM_) value followed by a progressive increase with the amount of CMC is observed.

Transverse relaxation times are related to the water molecule mobility within wet beads [23]. The T_2_ distribution can be exploited to estimate pore size distributions in porous cellulose [39] and nanofiber cellulose gels [40]. Johns and coworkers reported changes in T_2_ for regenerated bacterial cellulose hydrogels that were attributed to differences in pore size distribution (PSD) [24]. These cellulose beads displayed a bimodal distribution for T_2_, where values below 100 ms were ascribed to pores smaller than 100 nm, while values above this threshold were ascribed to pores between 100 and 1000 nm [24]. Here, the log-Gaussian distribution employed for the regenerated pure cellulose beads indicates a T_2LM_ of approximately 110 ms, indicating that the porous structure is predominantly composed of macropores while also containing mesoporosity.

In pure hydrophobic cellulose ether hydrogels, T_2_ decreases with an increasing solid content in the gels [37]. Figure 3b displays an initial drop in the average T_2_ values with the swelling. Therefore, it is necessary to differentiate between bound and free water fractions. Bound water is the combination of bound surface water and less-strongly bound water confined in meso-/macropores [41,42,43]. In highly swollen hydrogels and gels with large pores, the bound water fraction is lower, compared to in structures with smaller pores, meaning the signal arising from unbound water outweighs the contribution of the bound water signal in T_2_ [19]. In small pores, a higher specific pore confinement takes place and more bound water interacts with the surface, maximizing the influence of these “trapped” water molecules in the resulting T_2_ signal. In this case, the surface chemistry of the sorbent strongly affects bound water [19]. Altering the bead formulation changes the pore wall and the number of water-binding sites, offering the possibility of fine tuning the water diffusivity in the porous beads. In cellulose–CMC beads, this means that any changes in T_2_ have two underlying causes: swelling and surface chemistry. Strätz et al. demonstrated the importance of surface chemistry in cellulose sulfate-based gels: even small differences in the degree of substitution of oxidized cellulose sulfates radically change the T_2_ distributions. Gels with 0.20 and 0.28 aldehyde degrees of substitution possessed a wide bimodal distribution, while other gels gave rise to a narrow Gaussian distribution. The peak broadening was attributed to the heterogeneity of the sample. In the homogeneous systems, they did not observe a relationship between the aldehyde substitution and T_2_. The authors argue that the substitution only causes minor changes in the hydrogel density but does influence the cross-linking rate [44]. On the other hand, Agarwal and coworkers revealed that the carboxymethyl cellulose in microfibrillar cellulose suspensions disrupts the formation of strong bonds between fibers, improving their dispersion. As a consequence, the T_2_ was higher in samples that contained more CMC due to the increase in anionic groups, and the presence of fewer aggregate and fiber bundles in the suspension [45].

Prakobna and coworkers [46] designed mixed and core–shell systems of cellulose nanofibers and hemicellulose. It is interesting that the T_2_ of the neat cellulose fibers and core–shell structures displayed similar values, hinting at a comparable water molecule mobility. The T_2_ of the neat hemicellulose was higher, while the mixed system gave an intermediate value. In similar systems, Terenzi et al. [47] observed peak broadening, indicating inhomogeneous water regions, in a sample near 52% humidity, although this effect disappeared at 92% humidity. The three environments included bulk hemicellulose and both the coated and uncoated cellulose surface. They concluded that water mobility is faster at the coated interface compared to the uncoated fiber surface for lower relative humidity. The biocomposites at 92% relative humidity possessed a T_2_ between the ones from the neat fibers and hemicellulose. The widening observed in Figure 3a for the sample Cel30 hints that the CMC is less evenly distributed throughout the bead.

**Figure 4:**
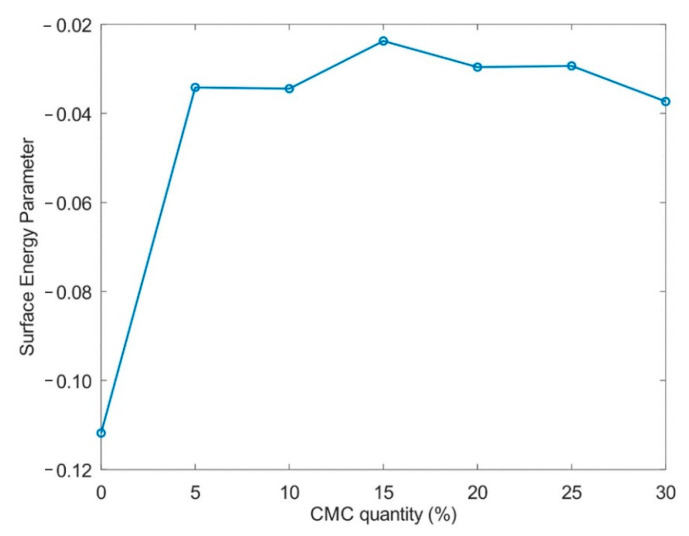
E_surf_ energy surface parameter as a function of the amount of CMC. This parameter shows the difference in energy interaction between the water molecules and the materials of different CMC contents. Upon adding the CMC, an initial increase in this parameter was detected, indicating a stronger interaction.

Figure 4 shows the energy interaction parameter E_surf_, calculated from the ratios of the average values of T_2_ and T_1_, according to Equation (3) (see the section Materials and Methods). This parameter is not affected by pore geometry and thus can be used to quantify changes related to interaction energy [48]. It is observed this parameter changes when the CMC is added to the beads and remains approximately constant with the increase in CMC content.

The E_surf_ parameter supports the interpretation of the results in the sense that the increase in T_1_ in the first step, from the pure cellulose to the 5% CMC, was caused by the increase in the energy interaction. CMC at 5% provides water-accessible carboxyl groups, enhancing the matrix–water interaction. Since the water interacts more with the material, causing a decrease in the mobility of the water molecules, we observe a drop in T_2_. With the increase in the CMC content, the energy remains constant. A combination of three factors contributes to a stable surface energy. First, the swelling of the samples raises the amount of unbound water in the pores, causing an increase in T_1_ and in T_2_. Next, a fraction of CMC could form an ordered supramolecular assembly, making the acidic groups inaccessible to water. Finally, the surface-bound CMC in the pores reaches saturation due to electrostatic repulsion, which causes the leaching of excess polyelectrolyte after washing.

Topochemical engineering aims for the rational fabrication of ordered 3D architectures. In this context, our results indicate that we can exploit the use of CMC to induce the ordered supramolecular assembly and increase the porosity of hydrogel beads. This concept can be further exploited in future work where multifunctional cellulose derivatives will be incorporated in bead fabrication.

## 3. Materials and Methods

### 3.1. Materials

Dissolving birch wood pulp was kindly provided by the Enocell Mill (Stora Enso, Uimaharju, Finland). Urea (99.5%) (Acros Organics, Geel, Belgium), Eurodenatured ethanol (>99%), hydrochloric acid (37%), sodium hydroxide and nitric acid (69%) were purchased from VWR (Leuven, Belgium). Carboxymethyl cellulose with a 250 kDa mass and DS of 0.9 was obtained from Acros Organics.

### 3.2. Bead Formulation

The pretreatment of the birch wood pulp was adapted from the procedure described by Trygg and coworkers [49,50] with a slight modification. In summary, 16 g of birch wood pulp was fibrillated and suspended in 400 mL of 92.5% ethanol containing 16 mL of HCl at 75 °C for 2 h. The pretreated pulp was washed extensively with distilled water, fibrillated using a kitchen grinder to facilitate dissolution, and air-dried.

Different mixtures of pretreated pulp and CMC were prepared so that the total moisture-free weight equaled 2.5 g (Table 2). The moisture content of the polysaccharides was calculated with a MA160 Moisture Analyzer (Sartorius, Göttingen, Germany). The resulting polysaccharides were added to 50 g of 7–12% sodium hydroxide–urea solution and dissolved at −10 °C under stirring. The polysaccharide solution was centrifuged before use to eliminate air bubbles. Beads were formed by the dropwise addition of the polymer solution through a 21 g × 4 ¾” Sterican^®^ hypodermic needle (B. Braun, Melsungen, Germany) in 400 mL of nitric acid (2 M) at 25 °C. After 1 h, the beads were extensively washed with distilled water and stored wet at 4 °C.

For critical point drying, the particles were dehydrated by a stepwise solvent exchange from water to ethanol, and then from ethanol to isopropanol. Liquid CO_2_ displaced the isopropanol inside the pores and was subsequently removed under supercritical conditions using an Autosamdri 815B critical point drying apparatus (Tousimis, Rockville, MD, USA).

### 3.3. Swelling Degree

Eighteen dry cellulose–CMC beads were weighed, followed by submersion in distilled water. After 24 h, excess surface water was removed from the swollen beads, and the particles were weighed again. The swelling degree was calculated as:

(1) $$S=\;\frac{W_{s}-\;W_{d}}{W_{d}},$$

with *W_S_* and *W_d_* being the weights of the swollen and dried beads. Eighteen beads/sample were measured to determine the maximal moisture uptake. The porosity was calculated similarly:

(2) $$\varepsilon_{p}\left(\%\right)=\frac{\frac{W_{s}-W_{d}}{\rho_{w}}}{\frac{W_{s}-W_{d}}{\rho_{w}}+\frac{W_{d}}{\rho_{c}}}\;\times 100,$$

where $\rho_{w}$ and $\rho_{c}\;$ represent the densities of water and cellulose, respectively [51].

### 3.4. SEM

Cross-sections of the wet hydrogels were taken using a scalpel and consequently dried by critical point drying. Pristine beads and their cross-sections were sputtered with chromium and imaged using a ZEISS Sigma Field Emission Scanning Electron Microscope (Oberkochen, Germany).

### 3.5. Specific Surface Area

The nitrogen sorption isotherms were measured at 77 K using a Mesopore 222 apparatus (3P instruments, Odelzhausen, Germany). The BET surface area was calculated using the instrument software (Version: 10.03.01, 3P instruments, Odelzhausen, Germany).

### 3.6. Low-Field ^1^H NMR Relaxometry

The NMR relaxometry experiments were performed using a portable NMR system, consisting of a LapNMR/Tecmag spectrometer and BT00250-AlphaSA/TOMCO amplifier, and a 0.3 T permanent magnet (corresponding to 13 MHz for ^1^H nuclei) and a radio-frequency probe. The magnet had a pseudo-Halbach design similar to the one published in [52] (courtesy of The RE Magnet Studio. Ltd., Nicosia, Cyprus). The details of this magnet assembly will be published elsewhere. The probe consisted of a solenoidal coil with 25 turns wound with 0.5 mm copper wire, having a 6.5 mm inner diameter and 12.4 mm length. For the tuning and matching of the probe, the solenoid was linked by a coaxial cable to an external box containing fixed and variable capacitors in parallel and series with the coil. The T_1_ and T_2_ relaxation times, from single swollen beads, were measured by CPMG (Carr–Purcell–Meiboom–Gill) and inversion-recovery (InvRec) experiments [53] after water excess removal [54]. The relaxometric data were fitted using the Inverse of Laplace Transform (ILT) software [55].

The radio-frequency pulse durations were 5.5 and 11 µs for the 90° and 180° pulses, respectively. The InvRec and CPMG experiments were performed with a repetition time of 20 s. Twenty distinct recovery times ranging from 100 µs to 20 s were used in the InvRec experiment. For each one, 8 scans were accumulated, resulting in a total acquisition time of 55 min per experiment. The CPMG experiments were recorded with an echo time equal to 200 µs. Only the central parts of the echoes, corresponding to 128 µs, were measured for each echo. In total, 3000 echoes were recorded and 64 scans were accumulated, resulting in 22 min of acquisition per experiment.

The individual NMR signals corresponding to each recovery time and echo were integrated, respectively, for the InvRec and CPMG experiments, and the corresponding curves were fitted using the ILT method, implemented on the Octave software, obtaining the distribution of the relaxation times. The average relaxation values in the logarithmic scale (T_1LM_ and T_2LM_) were calculated from the distributions and correlated with the CMC content.

The parameter of interaction energy was calculated from the ratio of *T*_2_ and *T*_1_, according to d’Agostino et al. [48]:

(3) $$E_{surf}\propto -\frac{T_{2}}{T_{1}}.$$

The errors presented in Figure 2b and Figure 3b were calculated through the results obtained by the log-Gaussian fittings of the *T*_1_ and *T*_2_ relaxation time distributions, according to the equation [23]:

(4) $$f\left(t\right)=\frac{A}{\sqrt{2\pi }C}e^{-\frac{1}{2}{\left(\frac{\log_{10}t-\log_{10}B}{C}\right)}^{2}\;}$$

with A being the amplitude, $\log_{10}B$ being the average value of the distribution and C being the variance of the distribution. Thus, C is the parameter that reflects the width of the distributions. In the linear scale, the parameter C is used to calculate the full width at half maximum (FWHM) as $FWHM=2\sqrt{2\ln2}C$. In this case, since the scale is logarithmic, uncertainties were calculated by $Err=C\log_{10}B$. The results of the fittings of T_1_ and T_2_ are presented in Table 3 and Table 4, respectively.

## 4. Conclusions

The incorporation of carboxymethylcellulose in cellulose beads offers new opportunities for topochemically engineering microspheres for dedicated applications. We demonstrate how the addition of even minor CMC amounts greatly enhances the water interaction with the bead interface. Water mobility is shown to improve in direct relation to the CMC content. The initial increase in NMR T_2_ relates to the modification of the chemical composition of the bead surface, creating a more hydrophilic interface that binds water more strongly. CMC also leads to changes in bead macrostructure, with the creation of larger inner pores. This increase in pore size, and the subsequent reduction in specific surface area, means that more unbound bulk-like water is detected inside the pores, which is reflected in the increase in T_2_ values. This increase in T_2_ is associated with enhanced water diffusivity. Cellulose–CMC microspheres with low CMC quantities could find applications such as use as moisture absorbers, as the particles possess a large surface area and bind water tightly. On the other hand, beads with higher CMC contents are attractive as a carrier material for catalysis due to their strong water interactions, while ensuring good water accessibility to potential catalytic sites.

## Figures and Tables

**Table 1:** Summary of dry polymer blends with a specified cellulose–carboxymethyl cellulose (CMC) % (*w*/*w*) giving rise to porous hydrogels beads. The average swelling degree (S), porosity (ε_p_) and BET (Brunauer–Emmett–Teller) area for the different beads for cellulose–CMC hybrid beads are reported with the standard deviations. Measurements of S and ε_p_ were performed on 18 beads/sample. Nitrogen adsorption isotherms were made in triplicate.

Sample	CMC (% *w*/*w*)	S Water	ε_p_ (%)	BET Area (m^2^/g)
Cel0	0	1.99 ± 0.50	74.23 ± 0.04	419 ± 17
Cel5	5	3.14 ± 0.51	82.18 ± 0.02	396 ± 24
Cel10	10	4.92 ± 0.60	87.92 ± 0.01	387 ± 20
Cel30	30	17.49 ± 4.18	96.16 ± 0.01	311 ± 3

**Table 2:** Quantities of cellulose and CMC added to NaOH–urea in order to fabricate a bead.

Sample	Cellulose (g)	CMC (g)
Cel0	2.500	0.000
Cel5	2.375	0.125
Cel10	2.250	0.250
Cel15 ^1^	2.125	0.375
Cel20 ^1^	2.000	0.500
Cel25 ^1^	1.875	0.625
Cel30	1.750	0.750

^1^ Samples measured only by NMR techniques.

**Table 3:** Parameters of the fittings of the T_1_ distributions, using a log-Gaussian function, presented in Equation (4).

CMC (%)	A	log_10_B	C	R^2^	Err
0	1270.0	1007.4	0.202	0.99734	203.5
5	1507.5	1091.2	0.202	0.99723	220.4
10	1879.2	1187.3	0.196	0.99123	232.7
15	1557.1	1321.0	0.165	0.99724	218.0
20	2232.8	1289.8	0.193	0.99729	248.9
25	2317.6	1483.8	0.189	0.99729	280.4
30	2730.6	1497.2	0.201	0.99736	300.9

**Table 4:** Parameters of the fittings of the T_2_ distributions, using a log-Gaussian function, presented in Equation (4).

CMC (%)	A	log_10_B	C	R^2^	Err
0	1837.9	110.6	0.151	0.99659	16.7
5	2132.2	35.3	0.175	0.99718	6.2
10	2725.0	38.3	0.181	0.99737	6.9
15	1250.7	31.9	0.105	0.99670	3.3
20	1790.7	38.7	0.150	0.99688	5.8
25	1848.9	43.3	0.149	0.99713	6.5
30	4577.3	56.5	0.219	0.99723	12.4

## Sample Availability

Samples can be provided upon request.
